# Study of the Relationship between ERCC1 Polymorphisms and Response to Platinum-based Chemotherapy in Iranian Patients with Colorectal and Gastric Cancers

**DOI:** 10.22037/ijpr.2019.1100827

**Published:** 2019

**Authors:** Mahdiye Abyarghamsari, Farshad Hosseini Shirazi, Maria Tavakoli-Ardakani, Hamid Rezvani, Hamid Reza Mirzaei, Jamshid Salamzadeh

**Affiliations:** a *Department of Clinical Pharmacy, School of Pharmacy, Shahid Beheshti University of Medial Sciences, Tehran, Iran. *; b *Pharmaceutical Sciences Research Center, Shahid Beheshti University of Medical Sciences, Tehran, Iran. *; c *Ayatollah Taleghani Hospital, Shahid Beheshti University of Medial Sciences, Tehran, Iran. *; d *Department of Radiation Oncology, Faculty of Medicine, Shahid Beheshti University of Medical Sciences, Tehran, Iran.*; e *Food Safety Research Center, Shahid Beheshti University of Medical Sciences, Tehran, Iran.*

**Keywords:** Oxaliplatin, Polymorphism, Colorectal cancer, Gastric cancer, ERCC1

## Abstract

This study was designed to evaluate the effect of excision repair cross complementing group 1 (ERCC1) rs11615 codon 118C/T gene polymorphisms on treatment outcomes in Iranian patients receiving oxaliplatin-based regimens for colorectal (CRC) and gastric cancers (GC). Patients, who were candidates to receive oxaliplatin-based chemotherapy, entered into the study. In 2-week intervals, the patients received combination regimen of oxaliplatin, fluorouracil, and leucovorin (FOLFOX) for 3 months. ERCC1 rs11615 codon 118C/T polymorphism was tested by restriction fragment length polymorphism polymerase chain reaction (RFLP-PCR) method using patients’ peripheral blood lymphocytes. The tumor response to chemotherapy was evaluated by examining the size of the tumor using CT scan. Association between response rates, according to the RECIST criteria, and patients’ genotypes was evaluated. Any relationship between response rate and possible explanatory factors was also determined. Overall, 40 patients (13 females (32.5%), and 27 males (67.5%)) enrolled in the study. Four patients (10.0%) carried the homo­zygous mutation (T/T genotype), ten patients (25.0%) were heterozygous (C/T genotype), and twenty-six patients (65%) were homo­zygous (C/C genotype). Response rate were 30.77%, 20.00%, and 0.00% for the genotypes C/C, C/T, and T/T, respectively. No significant association between response rate and genotypes was observed (*p* = 0.64). Patients with well- and moderately-differentiated histological grade of the tumor showed a better response rate (100.00% of 2 patients and 66.66% of 12 patients, respectively) compared to those with poorly differentiated (0.00% of 26 patients) histological grade (*p* < 0.001). Further multicenter studies are recommended to confirm conclusively our findings.

## Introduction

Gastrointestinal cancer, which includes cancers of the esophagus, gallbladder and biliary tract, liver, pancreas, stomach, small intestine, bowel (large intestine or colon and rectum), and anus is one of the most common causes of cancer deaths worldwide (1, 2). Each year approximately 3.25 million people are diagnosed with this illness (3). According to a report 2014 in Iranian population conducted by the World Health Organization (WHO), stomach cancer is the first common cancer in men and the third most common cancer in women. Furthermore, mortality rate of cancers involving colon, stomach and esophageal organs have substantial rates of 35.0% and 29.2% in men in women, respectively (2, 4 and 5). Moreover, according to a survey in 2016, stomach cancer is among the top ten causes of mortality among Iranian population (6). While, early detection and treatment of CRC decreases mortality, since patients usually ignore the early signs of the disease, most diagnoses are not timely. This leads to delays in diagnosis and, consequently, to treatment of the disease at advanced stages (7-9). Also, 40% to 50% of the patients who undergo potentially curative surgery alone, ultimately face relapse of the tumor and die of metastatic malignancy (10). The majority of the newly diagnosed patients, who are in the early stages of disease, can potentially be cured by a combination of surgery and chemotherapy; however, differences in clinical outcomes may exist (11).

Currently, the standard treatment of CRC and GC is fluorouracil plus leucovorin (folinic acid) that its efficacy is improved by addition of oxaliplatin (a platinum-based chemotherapeutic agent with a 1,2-diaminocyclohexane (DACH) carrier ligand) to this combination, particularly in patients with metastatic CRC (10). Clinical trials reveal that adding oxaliplatin to a regimen of fluorouracil combined with leucovorin, called FOLFOX regimen, produces a significant improvement in 3-year Disease-Free Survival (DFS) compared with a regimen consisting of fluorouracil and leucovorin administered without oxaliplatin (10, 12). In recent years, FOLFOX is the mainstay of CRC chemotherapeutics with a response rate of over 40%. The limited success could partly be due to resistance to conventional treatments (13). Unfortunately, in clinical practice, no explanatory factor is defined as a predictor for response to these treatment protocols (14).

Oxaliplatin has shown superior efficacy in comparison to other platinum based agents. Also, many tumor cell lines show fewer resistance against oxaliplatin compared to cisplatin and carboplatin (15, 16). Despite remarkable advances in cancer treatment, only those patients who respond appropriately to the dosage regimen, can benefit from the chemotherapy, while the non-responders mainly suffer from the toxicity and adverse reactions caused by cytotoxic anti-cancer drugs (17). Since, even at the same clinical stages, patients may show different responses and adverse drug reactions to chemotherapy, then, genetic variations are suspected as probable causes of heterogeneity of the outcomes (18). Currently, investigations regarding pharmacogenetical inter-individual differences are key steps towards personalized medicine (19, 20). Medication adherence in cancer patients is essential to obtain optimal health. On the other hand, it is proven that increment in the success rate of a treatment would also affect the success rate indirectly by increasing patient′s adherence to the treatment (21).

Oxaliplatin plays its role in tumor cell damage induction by DNA adduct which causes tumor cell apoptosis. Also, studies have shown that the DNA repair mechanism is an important genetic pathway in individualized sensitivity to chemotherapy (21, 22). While, there are several repair mechanisms in cells, the nucleotide excision repair (NER) pathway is the most versatile DNA repair mech­anism and is responsible for eliminating an extensive range of DNA lesions (18). ERCC1 and ERCC2 are two DNA repair genes on chromosome 19q13, that their products are important in NER and crucial for the removal of DNA adducts caused by platinum compounds (23, 24). As it is expected, ERCC1 plays a major role in the response to platinum-based therapies (14, 25 and 26) .With the same hypothesis, many studies have been conducted on the evaluation of the effects of polymorphism of ERCC1 on carcinogenesis and response to chemotherapy regimens (25, 27). It is possible that ERCC1 codon 118 polymorphism to be a biomarker for assessing the sensitivity to a platinum-based therapy. For this reason, several studies have assessed this relation in ovarian cancers, CRC, GC, and non-small cell lung cancers (25, 28-32). In fact, single nucleotide polymorphism at codon 118 (C→T) was reported to be associated with altered ERCC1 mRNA levels that can alter clinical outcomes of chemotherapy. However, the results about the relationship between ERCC1 codon 118 polymorphism, ERCC1 mRNA level, and platinum sensitivity are controversial (33).

The aim of the present study was to assess whether this polymorphism has any relationship with tumor response in patients with advanced or metastatic CRC and GC treated with the FOLFOX regimen. In addition, any relationship between possible explanatory factors includig demographic characteristics of the patients and their medical history with the ERCC1 codon 118 polymorphism were evaluated. 

## Experimental

*Patients and Methods*

From the year 2016 to 2018, 40 Iranian patients with non-resectable stage III and IV CRC and GC, who had received FOLFOX-6 as treatment, were recruited into the study. This study was conducted in a university affiliated hospital, the Ayatollah Taleghani Hospital located in Tehran, Iran. The inclusion criteria was adult patients (age >18 years), with a performance status less than or equal to two according to the Eastern Cooperative Oncology Group (ECOG) scale, and with an adequate bone marrow and renal function. Also, patients should have not been receiving any chemotherapy before enrollment in this study. Exclusion criteria were defined as co-existence of central nervous system metastases, serious or uncontrolled concurrent medical illness, and a history of other malignancies.

Informed consent was obtained from all patients. The protocol of the study was approved by the Ethics Committee of Shahid Beheshti University of Medical Science. All patients were asked to provide 2 mL of whole blood for genetic polymorphism testing. The demographic and clinical characteristics of the patients including histological classification of tumor were collected from their medical records. Histories of smoking and gastrointestinal cancers in the patient′s first degree family members were also reviewed.

*Chemotherapy Treatments and Response Criteria*

Oxaliplatin (85 mg/m^2^) in 500 mL of normal saline or dextrose was administered on day 1, by intravenous (I.V.) infusion over 1 h. On day 1, also, leucovorin (20 mg/m^2^) was administered as an I.V. bolus, immediately followed by 5-FU (400 mg/m^2^) given as 10-min I.V. bolus. This was followed by infusion of 5-FU (600 mg/m^2^) in 24-h. The patients received this regimen every two weeks. All patients had a computed tomography (CT) scan to measure lesion size at the time of the treatment initiation. The endpoint was the tumor response to chemotherapy after 12 weeks. This response was assessed according to the revised RECIST (34). An objective response to the treatment considered as “complete” (disappearance of the disease) or “partial” (at least 50% reduction in tumor load of the lesions) response. The patients with stable disease (≤25% progression, <50% shrinkage) or cancer progression (size enlargement >25% or appearance of new lesions) were classified as non-responder. 

*DNA Extraction and ERCC1 Codon 118 Polymorphism Genotyping*

Genomic DNA was isolated from 300 μL peripheral blood lymphocytes using a DNA extraction kit (Qiagen, Germany) according to the manufacturer′s instructions. The ERCC1 (C118T, rs11615) polymorphism was detected by the PCR- RFLP method. The PCR was done using 1.0 μL of genomic DNA, 12.5 μL of buffer PCR Master Mix (2X), and 1.0 μL of each primer, then diluted with DNase-RNase-free water for a final total volume of 25 μL. The PCR primers were 5’-GCA GAG CTC ACC TGA GGA AC-3’ and 5’-GAG GTG CAA GAA GAGGTG GA-3’. PCR conditions were 95 ºC for 5 min, followed by 40 cycles at 95 ºC for 1 min, 65 ºC for 1 min, 72 ºC for 1 min, and then 72 ºC for 10 min. The PCR products were digested by *BseMI* (*BsrDI*) (Thermo Scientific) restriction enzyme at 65 ºC for 4 h and were analyzed by 1% agarose gel electrophoresis in the presence of DNA stain. RFLP analysis of the resultant 199-bp fragment led to the identification of C/C (199 bp), C/T (199, 120, 79 bp), and T/T (120, 79 bp) genotypes.

*Statistical Analysis*

The Student’s *t*-test and the Fisher′s exact test were used to examine relationships between the clinico-pathological characteristics of the patients as well as the genetic polymorphisms of patients with their response to FOLFOX regimen. Significance level was *p*-value less than 0.05.

## Results and Discussion

Forty patients consisting of 13 females (32.5%) and 27 males (67.5%), with mean ± SD age of 58.08 ± 10.24 years, were entered the study. Frequency distribution of the patients based on their demographic and clinico-pathological characteristics is shown in Table 1.

*ERCC1 Genotypes*

The ERCC1 codon 118 genotypes were classified into homozygotes of CC, heterozygotes (CT), and homozygotes of TT. The frequencies of CC, CT, TT genotypes were 26 (65%), 10 (25%), and 4 (10%), respectively, as shown in Table 2.

*Relationship between ERCC1 polymorphism and response to chemotherapy*

Response to treatment was studied in 40 patients, of whom 10 (25.0%) were identified as responders and 30 (75.0%) as non-responders.TT allele of ERCC1 rs11615 was present in four patients which none of them responded to treatment. Moreover, CC and CT alleles were present in 26 and 10 patients, and response was seen in 8 and 2 patients, respectively. These results are summarized in Table 3.

Secondary analyses were done to explore any relationship between demographic variables, and clinico-pathological status of the patients with response to treatment. Results of these analyses are presented in Table 4.

As it is shown in the Table 4, there was an expectedly significant relationship (*p* < 0.001) between histological grade and the response to treatment, so that the patients with well- and moderately-differentiated histological grade of the tumor showed a better response rate (100.00% of 2 patients and 66.66% of 12 patients, respectively) compared to those with poorly differentiated histological grade (0.00% of 26 patients).

In accordance with the personalized medicine, along with the advancement in medical and pharmaceutical sciences, many prognostic and predictive biomarkers and pharmacogenomics testing have been investigated to individualize dosage regimen, maximize therapeutic effects, and minimize treatment toxicity. The goal of our study was to determine whether polymorphism at codon ERCC1 (C118T, rs11615) predicts the clinical outcome in advanced and metastatic CRC and GC patients receiving platinum-based chemotherapy. ERCC1 is a repairing enzyme encoded in chromosome 19q13.32, which consists of 10 exons. The scientific hypothesis of this study was that the DNA repair capacity of ERCC1 is a critical mechanism of resistance to platinum/5-FU-based drugs. 

Forty patients with CRC and GC have been studied in which the frequency of genotypes CC, CT, and TT were 65%, 25%, and 10%, respectively. Tumor response was assessed according to the RECIST criteria. Association between polymorphism and the response rate was evaluated using appropriate statistical methods, which was not significant. Furthermore, no significant associations were observed between polymorphism and the clinico-pathological variables, *i.e. *age, sex, cancer history, smoking, ECOG performance status, and except tumor histological grade. Nevertheless, the Fisher’s exact test with *p* < 0.001 showed that patients with well and moderate differentiated tumors responded to the FOLFOX regimen better than those patients with poorly differentiated tumors.

Several studies have been conducted on the frequency of the ERCC1 (C118T, rs11615) polymorphism in different populations, the effects of this polymorphism on protein expression, and the incidence of cancer as well as on resistance to chemotherapy regimens containing platinum drugs. In fact, in pharmacogenomics studies, while the effect of the gene in response is of considerable importance, its frequency in the target population should also be considered. The frequency of TT allele varies from 7% in the Korean population, to 48% among the German population. Although, the prevalence of this gene has not yet been studied in some populations, in this study, the frequency of this gene was about 10% in the Iranian population. Due to the small sample size, further investigations and screening studies are required for a more comprehensive conclusion. 

According to some studies, the codon 118 C/T polymorphism (rs11615) is associated with differential mRNA levels (26, 35). In fact, C→T polymorphism at codon 118 of ERCC1 results in the same amino acid asparagine; however, this transition converts a common codon usage (AAC) to an infrequent codon usage (AAT). At the same time, its frequency of use is reduced two-folds (36). Also, mRNA levels of ERCC1 and its association with resistance to chemotherapy in different cancers reveals that higher levels of the enzyme′s expression could decrease the response to the chemotherapy regimens containing platinum drugs (37-39). 

However, the association between this polymorphism and the level of mRNA and protein expression is still a matter of controversy. In some studies, it has been argued that this polymorphism improves the response to chemotherapy due to the reduced expression of the protein, while some other studies have denied these effects (27, 33, 35, 37 and 40-42). Therefore, association between the ERCC1 polymorphism and its mRNA levels needs further confirmation.

While *in-vitro *studies, using various human ovarian carcinoma cell lines, have confirmed that the C/C ERCC1 genotype is more effective in repairing platinum-DNA lesions, clinical researches on this subject have not yet reached a definitive conclusion. In our study, the ERCC1 codon 118 polymorphism was not associated with response to chemotherapy in advanced CRC and GC patients. 

This finding is in agreement with the results of some studies which had found that the ERCC1 codon 118 polymorphism was not correlated with the overall survival and response of advanced CRC and GC patients treated with platinum- based chemotherapy (23, 33). However, there were a number of studies describing that this polymorphism is associated with the response. They considered this mutation as a good predictor of the response to treatment which may contribute to the selection of the patients who would benefit from oxaliplatin-based chemotherapy in the future. 

In some articles, mutation reduces protein expression and reduces the ability to repair damage caused by the drug, resulting in patients with genotypes ERCC1 118T/T to have a better response to treatment (14). However, in other studies, the T allele was associated with a reduced response to chemotherapy and as a result, the genotype of ERCC1 118C/C seems to indicate better treatment outcome (17, 31 and 43). Moreover, these researches have emphasized the role of other confounding factors, such as alcohol consumption, cigarette smoking, and the stage of the disease that can affect treatment response. In our study, there was not any alcohol consumer among the patients, and the smoking and the stage of the disease did not show a relationship with the response rate. At any rate, further extensive studies are needed to promote the appropriateness of treatment options in this area.

Our study had two major limitations. First, since the number of CRC and GC patients was relatively low, the statistical power of the analyses to identify an association between the ERCC1 polymorphisms and clinical outcome of the treatment is limited. Secondly, in this study we only examined the role of one common SNP in the ERCC1 gene. Since DNA repair is a complex collection of processes, many DNA repair genes may be involved and may confuse the results. Other functional SNPs in the DNA repair system may influence the survival of the patients with CRC and GC, which must be investigated in further studies. Finally, practical data supporting the association between the ERCC1 polymorphism and its activity are still controversial and inadequate.

**Table 1:** Clinical and Demographic Characteristics of Included Subjects (N = 40)

**Age (mean ± SD)**	**Range 31-78 years (58.08 ± 10.24)**	
Gender	Male	27 (67.5%)
	Female	13 (32.5%)
Histology	Squamous Cell Carcinoma	2 (5%)
	Adenocarcinoma	38 (95%)
Smoking history	yes	5 (12.5%)
	no	35 (87.5%)
Family History of CRC and GC	Yes	5 (12.5%)
	No	35 (87.5%)
Differentiation Status	Well	2 (5%)
	Moderate	12(30%)
	Poor	26 (65%)
ECOG Performance Status	0-1	38 (95%)
	2	2 (5%)
Tumor Staging	III	2 (5%)
	IV	38 (95%)
Tumor Location	Gastric	7 (17.5%)
	Colon/Rectum	33 (82.5%)

ECOG: Eastern Cooperative Oncology Group; CRC: Colorectal Cancer.

**Table 2:** Frequency of the Genotypes ERCC1 codon 118

**Genotype**	**CC**	**CT**	**TT**
Distribution (N = 40)	26 (65%)	10 (25%)	4 (10%)

ERCC1: excision repair cross complementing group 1.

**Table 3:** Association between the ERCC1codon118 Polymorphism and the Response to Chemotherapy

**Genotype**	**Total**	**TT**	**CT**	**CC**
Yes	10 (25.00%)	0 (00.00%)	2 (20.00%)	8 (30.77%)
Response				
No	30 (75.00%)	4 (100.00%)	8 (80.00%)	18 (69.23%)
Total	40 (100.00%)	4 (100.00%)	10 (100.00%)	26 (100.00%)

ERCC1: excision repair cross complementing group 1.

**Table 4:** Association between demographic, and clinico-pathological variables with the response to chemotherapy

		**Response**		**Total**	***p*** **-Value (0.95)**
		**No**	**Yes**		
	Female	11 (84.62%)	2 (15.38%)	13 (32.5%)	
Gender					0.45
	Male	19 (70.37%)	8 (29.63%)	27 (67.5%)	
	<58	14 (70.00%)	6 (30.00%)	20 (50%)	
Age					0.25
	≥58	16 (80.00%)	4 (20.00%)	20 (50%)	
	Negative	25 (71.43%)	10 (28.57%)	35 (87.5%)	
Family history					0.31
	Positive	5 (100.00%)	0 (0.00%)	5 (12.5%)	
	No	25 (71.43%)	10 (28.57%)	35 (87.5%)	
Smoking					0.31
	Yes	5 (100.00%)	0 (0.00%)	5 (12.5%)	
	Poorly differentiated	26 (100.00%)	0 (0.00%)	26 (65%)	
Histological grade	Moderately differentiated	4 (33.33%)	8 (66.67%)	12 (30%)	0.001
	Well differentiated	0 (0.00%)	2 (100.00%)	2 (5%)	

## Conclusion

Published data on ERCC1 polymorphisms and efficacy of oxaliplatin are inconsistent. Generally, ERCC1 SNPs cannot be considered as standard marker of survival or response in oxaliplatin treated patients. One reason for this may be lack of a standardized methodology and polymorphism assays. In this study, no relationship between the polymorphisms in ERCC1 (rs11615) and response to chemotherapy of advanced and metastatic CRC and GC patients were found; however, the trend of response rate was in favor of the patients with C/C allele. This uncertainty might be due to insufficient sample size of the present study. 

Future studies, preferably within the setting of larger multicenter randomized prospective control trials with longer follow up of response (with overall response rate and progression-free survival (PFS)), should try to determine how accurately these markers will predict the final outcomes. On the other hand, the impact of confounding factors such as sex, age, TNM stage, smoking, and alcohol drinking habits, which would make the results masked, must be reduced or deleted. These approaches would help to design clinical studies with more precise results. This will, hopefully, allow the care providers to present a high-quality treatment with fewer toxicity for cancer patients in the scope of individualized medicine. 
